# Supplementary material for: Proof of concept: targeted protein degradation of the stress granules component G3BP1 as an antiviral strategy against norovirus infection
Source: Antimicrob Agents Chemother. 2026 Jan 26;70(3):e01118-25. doi: 10.1128/aac.01118-25 (PMC12959154; doi:10.1128/aac.01118-25)
Supplement: Supplemental figures — Fig. S1 and Fig. S2. [file aac.01118-25-s0001.docx]

**Supplementary data**

**
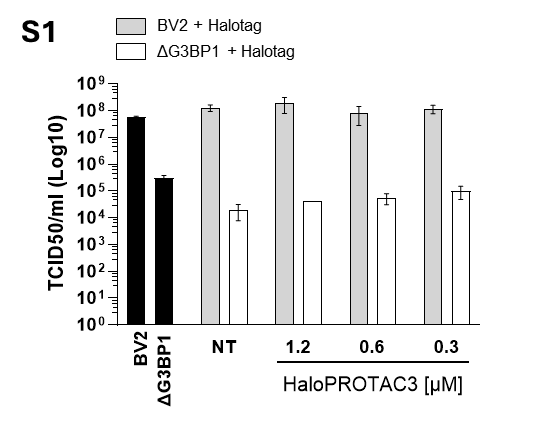
**

**Figure S1.** Extracellular infectious virus yield quantified by TCID_50_ in BV2 cells from supernatants of BV2, ΔG3BP1, BV2 + Halotag or ΔG3BP1 + Halotag cells infected with MOI 1 TCID_50_/cell and treated with the indicated concentrations of HaloPROTAC3.

**
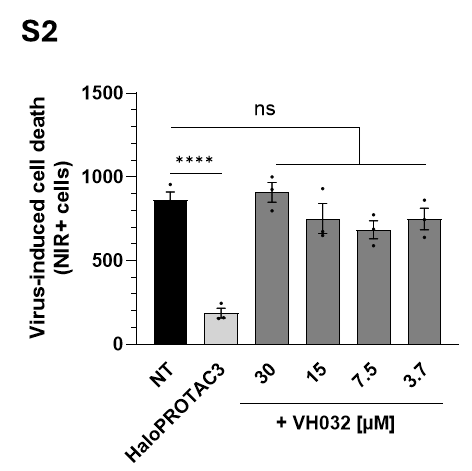
**

**Figure S2.** BV2 ΔG3BP1 + HaloG3BP1 were treated with either HaloPROTAC3 or VH032 at the indicated concentrations and infected with MNV1 at MOI 1 TCID_50_/cell. Infection-induced cell death was detected using the NIR dye measured in the live cell imaging Incucyte system. The quantification of the total number of NIR + cells after 24 h.p.i. is shown. Data is expressed as mean ± standard error of the mean with ^∗∗∗∗^ indicating *P* ≤ 0.0001. ‘ns’ denotes non-significant.
